# Letter from Dr. F. T. Paine on Electrical Treatment of Diseases

**Published:** 1887-10

**Authors:** 


### CORRESPONDENCE.

### LETTER FROM DR. F. T. PAINE,

ON AN INTERESTING AND IMPORTANT SUBJECT.

COMANCHE, TEXAS, September 28, 1887.

*Dr. F. E. Daniel.*

DEAR SIR: In your notice of my paper in the Transactions, you very truly say, "if future observations shall confirm the statements here recorded by Dr. Paine, there is no estimating the value of electricity as a remedial agent," etc.

Now, if I should leave these experiences and observations where they are, unrecognized by the profession, it may be a century before another will rise up, willing to take my place, and face the derision and obloquy that I have met, as unaided,—struggling against poverty, unpaid, and many times even *unthanked*, I have prosecuted these studies, solely for the benefit of suffering humanity, and without even the *prospect* of pecuniary advantage.

In view of this, I have a proposition to submit to you, and through your excellent JOURNAL to the profession.

I am anxious to place these discoveries in the hands of our profession, while I may, and as I observe that the Austin District Medical Society is to meet in your city on the 8th of December, next, I would be pleased to celebrate the 73rd anniversary of my birth * by reading a paper (prepared especially for that occasion) on the subject of neuralgia, with the differential diagnosis of neuralgia, and other painful diseases ; making the subject so plain that "he that runs may read, and not err therein, (provided he uses electricity,)tho' he be a fool." This paper or papers I shall defend publicly, or demonstrate the treatment upon the living subject (if subjects can be procured). On the subject of diseases of females, I shall be prepared to demonstrate all I have ever written, if subjects can be procured. I propose to demonstrate the painlessness of all proper applications of Farradic electricity, even in the most painful and irritable diseases.

If the profession wish the demonstration of the truths I have discovered, a committee from the Austin District Medical Society can have my services to elucidate the subject just as far as I possibly can. I would suggest that the society might, and doubtless will, enlist the co-operation and interest of the medical gentlemen of the State Lunatic Asylum, who could furnish a vast amount of material for the study of the effects of electricity on mental diseases and diseases of females.

If these propositions meet the approbation of the Board of Censors of the Austin District Medical Society, an early invitation to meet them at a time to suit their convenience will be agreeable to me.

Very truly yours, etc.,

F. T. PAINE, M. D.

*The 8th of December will be my 73rd birthday.

[It will be remembered that Dr. Paine pointed out, in the January, '86, No. of this JOURNAL, Vol. 1, No. 7, ("Revalations by Electricity in Rheumatism and Neuralgia,") that the most painful parts in cases of acute and chronic rheumatism, are *perfectly insensible* to the electric current, as much so, he says, as if the nerve were dead; and, indeed, the Doctor says the *nerve is* dead; and that in certain female diseases the mammæ and the genitalia are equally anæsthetic (electrically). He relates that in some cases an electrode may be introduced into the vagina, not only without pain, but without sensation, which, in another person, would seem as if a red-hot iron had been thrust into the part. These are highly interesting facts, discovered, as Dr. Paine claims, by himself, and as important as they are interesting. We shall be glad to witness the demonstrations, and sincerely hope the Austin District Medical Society will invite the Doctor to be with them at the approaching meeting, and prepare the material for the demonstrations. We understand that Dr. Paine proposes to map out, on a diagram, certain points on the surface of individuals suffering with perversion of the sexual sense,—which are invariably electrically anæsthetic,—and to demonstrate his theory that there is a relation between this condition of the nerve centers and certain heretofore obscure and intractable female maladies.

Dr. Paine has spent a lifetime in the practice of medicine, and, although he has lived out his scriptural measure of years, he is still actively engaged in the good work. His long and close observation of clinical phenomena, his advanced age, and his position in the profession, as well as his character and standing as a gentleman, a Christian, and a practitioner, entitle his words to respectful attention and weighty consideration. We repeat what we said before, (not that we question for a moment either his veracity or the correctness of his observations) that if future experiments and observations shall tally with his past experience, and confirm his views, electricity is destined to become the most powerful and most reliable weapon in the physician's armamentarium.

